# Hyperspectral reconstruction of white light endoscopy images for enhanced segmentation of early esophageal lesions in patients

**DOI:** 10.1016/j.isci.2026.115460

**Published:** 2026-03-21

**Authors:** Yao-Kuang Wang, Kun-Hua Lee, Chun-Hsien Su, Chia-Ling Chen, Wei-Chung Chen, I-Chen Wu, Cheng-Yi Wang, Hsiang-Chen Wang

**Affiliations:** 1 Graduate Institute of Clinical Medicine, College of Medicine, Kaohsiung Medical University, No.100, Shiquan 1st Road, Sanmin District, Kaohsiung City 80756, Taiwan; 2 Division of Gastroenterology, Department of Internal Medicine, Kaohsiung Medical University Hospital, Kaohsiung Medical University, No.100, Shiquan 1st Road, Sanmin District, Kaohsiung City 80756, Taiwan; 3 Department of Medicine, Faculty of Medicine, College of Medicine, Kaohsiung Medical University, No.100, Shiquan 1st Road, Sanmin District, Kaohsiung City 80756, Taiwan; 4 Department of Trauma, Changhua Christian Hospital, No.135, Nanxiao Street, Changhua City, Changhua 50006, Taiwan; 5 Department of Mechanical Engineering, National Chung Cheng University, 168, University Road, Min Hsiung, Chia Yi 62102, Taiwan; 6 Biomedical Artificial Intelligence Academy, Kaohsiung Medical University, No.100, Shiquan 1st Road, Sanmin District, Kaohsiung City 80756, Taiwan; 7 Department of Gastroenterology, Kaohsiung Armed Forces General Hospital, 2, Zhongzheng 1st. Road, Kaohsiung City 80284, Taiwan; 8 Department Director of Technology Development, Hitspectra Intelligent Technology Co., Ltd., Kaohsiung 80661, Taiwan

**Keywords:** health sciences, medicine, health technology, diagnostic technique in health technology

## Abstract

Early esophageal squamous neoplasia is difficult to detect under conventional white light imaging due to subtle mucosal and vascular changes. This study utilizes a spectrum-aided vision enhancement (SAVE) framework to computationally reconstruct 401-band hyperspectral information from standard RGB endoscopy images. By integrating these virtual spectral features with a U-Net semantic segmentation model, the approach enhances lesion boundary delineation without requiring specialized hyperspectral hardware. Evaluation on 531 clinical images demonstrates that models using virtual spectral data achieve a mean intersection over union of 74.3%, outperforming conventional white light and narrow-band imaging baselines. Cross-center validation further confirms the generalizability of this model-agnostic enhancement across different endoscopy systems. These findings indicate that virtual hyperspectral reconstruction provides a feasible strategy for enriching diagnostic features in routine clinical workflows. This approach offers a scalable tool for improving the computer-aided detection of early-stage gastrointestinal malignancies.

## Highlights

• SAVE reconstructs pseudo-hyperspectral data from standard RGB endoscopy images
• SAVE-enhanced U-Net improves lesion segmentation mIoU from 69.0% to 74.3%
• Cross-center validation confirms robust performance across endoscope models
• Virtual hyperspectral imaging provides a scalable tool for early cancer detection

## Graphical abstract

**Figure:**
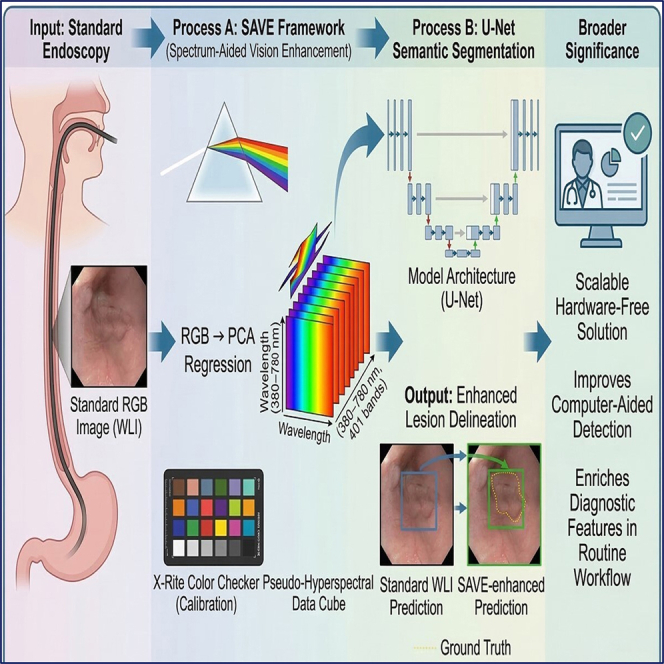

## Introduction

Esophageal cancer is recognized globally as a highly lethal malignancy.^1^^,^^2^^,^^3^^,^^4^^,^^5^^,^^6^ According to global cancer statistics, it ranks among the top 10 causes of cancer-related mortality.^7^^,^^8^ A majority of patients are diagnosed at an advanced stage, primarily due to the subtle and nonspecific appearance of early lesions under endoscopy. These early-stage abnormalities often manifest as minor vascular changes or mild mucosal inflammation only, making detection heavily reliant on the subjective judgment of experienced endoscopists and susceptible to interobserver variability.^9^^,^^10^ The sensitivity of conventional white-light imaging (WLI) for early lesion detection is relatively low, ranging from 65% to 72%.^11^ Although iodine staining can enhance detection,^12^ it is invasive and may trigger allergic reactions. Narrow-band imaging (NBI), which enhances vascular contrast by using 415 nm and 540 nm wavelength bands, can improve diagnostic accuracy to approximately 85%.^13^ However, the risk of missed diagnoses remains between 15% and 20% due to high equipment costs and false negatives associated with indistinct lesion boundaries.^14^

Most patients with esophageal cancer remain asymptomatic during the early stages, leading to missed opportunities for timely diagnosis. Detection often occurs only when the advanced tumor obstructs more than half of the esophageal lumen, resulting in dysphagia, at which point lymph node metastasis or other complications may already be present, complicating treatment.^15^^,^^16^^,^^17^^,^^18^ Early-stage diagnosis is challenging due to the subtle visual presentation of inflammation in endoscopic images, typically limited to minor vascular changes that can be accurately identified by experienced endoscopists only. Although misinterpretations are possible, diagnostic accuracy has been improved using iodine staining and NBI, both of which enhance lesion visibility.^9^^,^^19^^,^^20^^,^^21^^,^^22^^,^^23^

Although several studies have applied semantic segmentation techniques to the analysis of esophageal cancer images,^24^^,^^25^^,^^26^^,^^27^^,^^28^^,^^29^ most are still limited to RGB images. The spectral information provided by the three channels of RGB is insufficient to capture subtle optical features in lesion regions, thereby reducing the ability to identify early-stage lesions. In the present study, a method that utilizes a hyperspectral imaging (HSI) system to convert conventional WLI images into hyperspectral representations that highlight lesion features more clearly is proposed. By integrating the HSI system with a deep learning-based semantic segmentation model, U-Net, this study aimed to achieve more precise lesion localization and facilitate early detection in clinical settings.^30^^,^^31^^,^^32^^,^^33^

HSI can capture spectral features across 401 continuous bands within the 380–780 nm range, providing an “optical fingerprint” of tissue. This approach belongs to the broader field of biomedical spectral imaging, in which conventional imaging is integrated with spectroscopy to obtain spatially resolved spectral signatures for tissue characterization, as comprehensively reviewed by Li et al.^34^ Compared with RGB images, HSI offers enhanced resolution of mucosal layers and subtle variations in blood oxygenation and metabolism.^35^^,^^36^ International studies have demonstrated its potential in early cancer diagnosis, showing a sensitivity of up to 89.3% for detecting precancerous gastric lesions.^37^ In parallel with these advances, recent work in gastrointestinal imaging has increasingly focused on spectral and hyperspectral endoscopy systems designed to overcome the limitations of WLI and fixed-band modalities such as NBI. Early multispectral platforms, including the endoscopic polarized scanning spectroscopy system for Barrett’s esophagus proposed by,^38^ showed that wavelength-resolved reflectance can reveal dysplastic mucosa that appears flat under both high-definition WLI and NBI. More recent hyperspectral endoscopic systems, such as the HySE platform developed and the fiber-bundle spectral endoscope, demonstrated the technical feasibility of *in vivo* GI HSI and achieved approximately 85% accuracy in differentiating Barrett’s neoplasia from non-dysplastic mucosa.^39^^,^^40^ Computational approaches have also emerged that derive virtual spectral information from RGB endoscopy, including our earlier spectrum aided vision enhancer (SAVE) method and CycleGAN-based WLI-to-synthetic-NBI conversion frameworks, both of which generate pseudo-spectral images that emulate the contrast enhancements of hardware-based systems.^41^^,^^42^ Additionally, deep learning models integrating HSI-derived pixel spectra by our group have shown superior detection accuracy for early esophageal squamous neoplasia compared with RGB-only inputs.^31^ Recently, advanced deep learning frameworks have been proposed to tackle the complexities of medical hyperspectral data. For instance, Qin et al. introduced UFPF, a universal feature perception framework that leverages a hierarchical “corner-to-center” Mamba structure to effectively capture spatial-spectral dependencies in microscopic images.^43^ In the domain of semi-supervised learning, ACCL-CINet was proposed to enhance segmentation performance by integrating adversarial consistency constraint learning with a cross-indication network.^44^ These studies demonstrate the growing importance of sophisticated feature fusion strategies in medical imaging. However, while these methods focus on optimizing network architectures to process available hyperspectral data, they typically rely on specialized acquisition hardware. In contrast, our SAVE framework addresses the accessibility of spectral data in routine endoscopy. Instead of requiring hyperspectral sensors, SAVE focuses on the physics-based reconstruction of spectral information directly from standard RGB images, thereby bridging the gap between advanced spectral analysis and standard clinical equipment.

These developments collectively underscore the growing recognition that spectral information, whether obtained directly via HSI hardware or reconstructed computationally, can enhance mucosal contrast and improve computer-aided detection, yet existing methods largely focus on classification or visual enhancement rather than achieving end-to-end, pixel-level segmentation tailored specifically for early esophageal cancer.

Building upon this evidence, a refined version of the previously developed SAVE method was constructed in the present study to convert WLI images into high-contrast hyperspectral representations that simulate NBI-like enhancement while preserving fine mucosal detail. The converted high-contrast images were then analyzed using a U-Net semantic segmentation model to overcome the limitations of current technologies. Deep learning applications in cancer detection have advanced rapidly,^45^ and the U-Net model, with its encoder-decoder architecture,^46^ enables the preservation of global semantic context and local lesion detail. Recent studies have shown that AI-assisted endoscopic diagnosis can improve accuracy by 12%–18%,^47^ although most existing models remain limited by the insufficient spectral information of RGB images. The present study is the first to integrate HSI spectral features with U-Net, offering a more accurate solution for the early detection of esophageal cancer.

## Results

**Figure 1:**
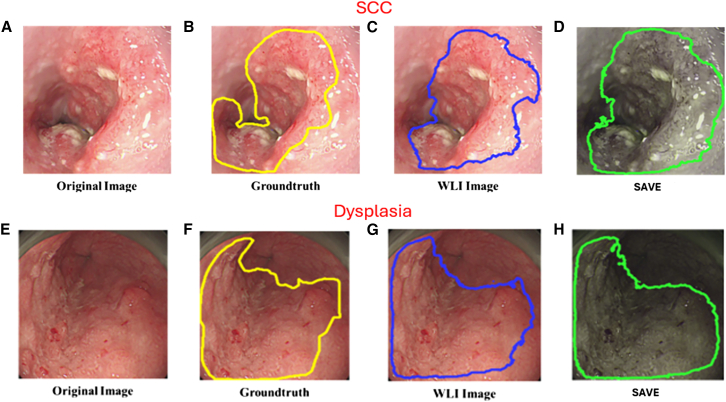
Detection of early esophageal neoplasia using white light imaging in patients (A–D) Prediction results for squamous cell carcinoma (SCC). (A) Original white-light imaging (WLI) image. (B) Manually annotated ground truth (yellow box). (C) Prediction from the WLI-based model (blue box). (D) Prediction from the SAVE model (green box). (E–H) Prediction results for dysplasia lesions. (E) Original WLI image. (F) Ground truth (yellow box). (G) WLI-based model prediction (blue box). (H) SAVE model prediction (green box). Yellow, blue, and green bounding boxes represent the ground truth, WLI-model predictions, and SAVE-model predictions, respectively.

**Figure 2:**
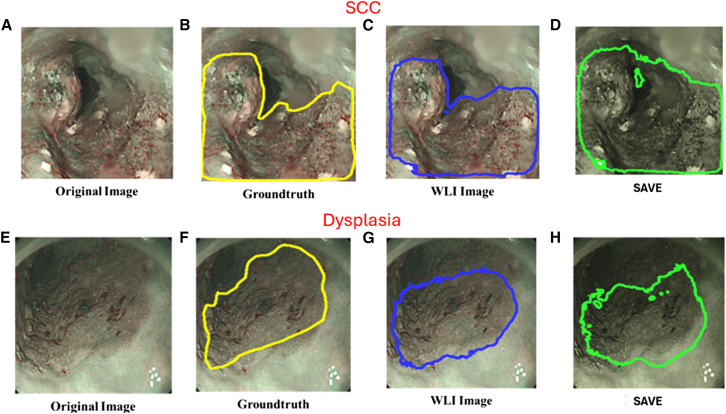
Results of early esophageal cancer detected using narrow-band imaging (NBI) (A) Original NBI image of squamous cell carcinoma (SCC) case. (B) Manually annotated ground truth bounding box of SCC. (C) Bounding box of SCC predicted using the NBI detection model. (D) Bounding box of SCC predicted using the SAVE detection model. (E) Original NBI image of dysplasia case. (F) Manually annotated ground truth bounding box of dysplasia. (G) Bounding box of dysplasia predicted using the NBI detection model. (H) Bounding box of dysplasia predicted using the SAVE detection model.

This study utilized 320 WLI images and 211 NBI images of early esophageal cancer to construct prediction models for RGB and SAVE-transformed images. A total of three models were developed and evaluated. The true lesion regions were marked with yellow bounding boxes, predictions from the WLI model were shown in blue, and predictions from the SAVE model were represented in green. Figures 1A–1D present the prediction results of the WLI-based and SAVE-based models for SCC, and Figures 1E–1H display the prediction results for dysplasia lesions using both models. Figure 1A shows the original WLI image of an SCC lesion, and Figure 1B presents the corresponding ground truth annotation in a yellow bounding box. Figure 1C depicts the prediction result from the WLI-based model, with the lesion boundary marked in blue. By contrast, Figure 1D illustrates the prediction by the SAVE model, with a green bounding box more clearly outlining the lesion and more closely aligning with the ground truth compared to the WLI model. Similarly, Figure 1E shows the original image of a dysplasia case, followed by the ground truth annotation in Figure 1F in yellow, the prediction from the WLI model in Figure 1G in blue, and the prediction from the SAVE model in Figure 1H in green. The results demonstrate that the SAVE model significantly enhances image contrast and improves prediction accuracy, particularly in dysplasia cases where the lesion boundaries are ambiguous and difficult to identify, thus highlighting its potential in early lesion detection. Figures 2A–2D show the SCC detection results of the models trained on NBI and SAVE images, and Figures 2E–2H present the dysplasia detection outcomes by using the same two models. Specifically, Figure 2A is an unannotated original NBI image of an SCC lesion. Figure 2B shows the manually annotated lesion boundaries in yellow. Figure 2C provides the prediction result from the model trained on original NBI images, represented in blue. Figure 2D shows the prediction result from the model trained on SAVE-transformed images, where the green bounding box demonstrates improved precision in delineating the lesion boundary. Figure 2E displays an original NBI image of a dysplasia case, which is typically difficult to identify due to unclear visual features. The ground truth annotation is shown in Figure 2F in yellow, and the NBI model’s prediction in Figure 2G shows partial deviation from the actual lesion area. In comparison, Figure 2H presents the prediction from the SAVE-based model, which more accurately aligns with the ground truth, especially in the fine boundary areas, illustrating its effectiveness in enhancing lesion detection accuracy.

Multiple performance metrics were employed, including intersection over union (IoU) and sensitivity for category-specific analysis, along with Matthews correlation coefficient (MCC) for assessing the overall model performance, to evaluate the experimental results. The IoU metric serves as a combined indicator of classification and localization capabilities, and sensitivity reflects the model’s ability to correctly detect lesion regions. As object detection tasks require evaluation of classification and localization and often include a background class, accuracy alone is insufficient for a comprehensive assessment. Therefore, the mean IoU (mIoU), which averages the IoU across all classes, was used as a representative measure of overall detection performance. This metric integrates class prediction and spatial localization, and it is one of the most widely used indicators in object detection. The statistical results are summarized in Table 1. For the white-light endoscopic esophageal cancer detection model, a total of 411 WLI and NBI images were collected as the training set, and 120 images were used as the test set. The model was trained with 50 epochs and a batch size of 8. After 50 epochs of training, evaluation on the test set yielded the following performance metrics: an mIoU of 71.0%, a mean pixel accuracy (mPA) of 83.9%, an overall accuracy of 83.9%, a precision of 74.9%, a recall of 95.9%, a specificity of 77.4%, an F1-score of 82.1%, and an MCC of 70.8%.

**Table 1:** Performance metrics for white light and SAVE models on the internal test set

Methods	mIOU (%)	mPA (%)	Precision (%)	Recall (%)	Specificity (%)	F1-score (%)	MCC (%)
WLI	69.0 ± 1.2	83.9 ± 1.3	74.9 ± 1.3	94.9 ± 1.3	77.4 ± 1.2	83.7 ± 1.4	70.8 ± 1.4
SAVE	74.3 ± 1.1	85.1 ± 1.2	80.5 ± 1.2	91.2 ± 1.2	79.9 ± 1.2	85.5 ± 1.3	71.2 ± 1.2

Performance evaluation of the U-Net model using conventional WLI and SAVE-enhanced inputs. Metrics include mean intersection over union (mIoU), mean pixel accuracy (mPA), precision, recall, specificity, F1-score, and Matthews correlation coefficient (MCC). Data are presented as mean ± standard deviation (SD). The test set consists of 120 independent endoscopic images derived from 25 patients.

For the HSI-U-Net model, training was conducted using 50 epochs with a batch size of 8. After 50 epochs were completed, the evaluation on the test set yielded the following results: an mIoU of 74.3%, an mPA of 85.1%, an overall accuracy of 85.1%, a precision of 80.5%, a recall of 91.2%, a specificity of 79.9%, an F1-score of 84.6%, and an MCC of 71.2%. Among the evaluation metrics, the mIoU score reached an average of 74.3%. From a clinical perspective, an mIoU exceeding 70% indicates a high degree of overlap between the predicted lesion area and the ground truth annotations. Such a level of agreement suggests that the boundary localization error is likely within 2 mm, based on typical clinical image resolution and endoscopic image dimensions. Such precision demonstrates practical diagnostic value. For early lesion detection, ensuring the model’s predicted boundaries fall within ±2 mm of the actual lesion margins is critical for accurate biopsy and resection site localization.

The model achieved a recall of 91.2% under the SAVE-enhanced images, indicating its strong sensitivity and ability to detect most lesion regions. However, the corresponding precision was only 80.5%, suggesting that while the model successfully identifies a majority of abnormal areas, it also tends to over-segment and generate false positive regions. This trade-off is common in early-stage detection tasks—high recall helps minimize missed diagnoses but may increase the false positive rate, potentially complicating clinical interpretation. Post-processing techniques can be applied for optimization to address this issue. Adjusting the thresholds of non-maximum suppression (NMS) or incorporating conditional random fields can effectively filter out small or low-intensity false-positive regions. Integrating uncertainty estimation or confidence mapping may help correct predictions with low confidence scores. Employing ensemble averaging of multiple models can enhance prediction stability and improve overall accuracy.

### Generalizability of SAVE across state-of-the-art architectures

To validate the robustness and generalizability of the SAVE framework beyond the standard U-Net baseline, we extended our evaluation to include two state-of-the-art segmentation architectures: DeepLabv3+ (representing advanced CNN-based encoders) and SegFormer (representing Transformer-based architectures). As presented in Table 2, the integration of SAVE-enhanced images yielded consistent performance improvements across all tested network architectures. Specifically, for DeepLabv3+, the use of SAVE inputs improved the mIoU from 68.2% (WLI) to 72.3%, accompanied by a notable increase in precision from 72.3% to 80.1%. Similarly, the Transformer-based SegFormer model demonstrated improved performance with SAVE, increasing mIoU from 70.5% to 73.1% and precision from 73.6% to 80.4%. Interestingly, the baseline U-Net model achieved the highest overall performance with SAVE (mIoU 74.3%), suggesting that for this specific dataset size and task, the U-Net architecture remains highly effective when coupled with spectrally enhanced features. Across all models, the most significant gain was observed in Precision, with an average increase of approximately 7%. This indicates that the spectral contrast provided by SAVE effectively suppresses false positives in the background, allowing the models to delineate lesion boundaries more accurately than with standard WLI.

**Table 2:** Performance comparison of different segmentation networks (U-Net, DeepLabv3+, and SegFormer) using standard white light imaging (WLI) versus SAVE-enhanced inputs

Model Architecture	Input Modality	mIoU (%)	Precision (%)	Recall (%)
U-Net (Baseline)	WLI	69.0	74.9	95.9
U-Net (Baseline)	SAVE	74.3	80.5	91.2
DeepLabv3+	WLI	68.2	72.3	93.5
DeepLabv3+	SAVE	72.3	80.1	94.4
SegFormer	WLI	70.5	73.6	95.2
SegFormer	SAVE	73.1	80.4	96.0

### Comparison with standard contrast enhancement

To investigate whether the performance improvement of SAVE stems from spectral reconstruction or simple contrast enhancement, we compared our method with contrast limited adaptive histogram equalization (CLAHE), a standard image processing technique. As shown in Table 3, while CLAHE provided a marginal improvement over the WLI baseline (mIoU: 70.5% vs. 69.0%), the SAVE framework achieved a significantly higher mIoU of 74.3%. Furthermore, SAVE outperformed CLAHE in Precision (80.5% vs. 76.0%), suggesting that CLAHE enhances noise and background textures indiscriminately, whereas SAVE selectively recovers lesion-specific spectral features. This confirms the necessity of the spectral reconstruction step for accurate lesion segmentation.

**Table 3:** Ablation study comparing SAVE with standard contrast enhancement (CLAHE)

Method	mIoU (%)	Precision (%)	Recall (%)
WLI (Baseline)	69.0	74.9	95.9
WLI + CLAHE	70.5	76.0	94.5
SAVE	74.3	80.5	91.2

### External validation performance

External validation results are summarized in Table 4. In the NTUH dataset, the images generated by the SAVE frame outperformed traditional WLI images in both mIoU (72.2% ± 1.1%) and precision (77.1% ± 1.2%). This indicates that the SAVE frame can successfully capture the spectral characteristics of lesions in different endoscope models (Q260/H260).

**Table 4:** Performance comparison of lesion segmentation using standard WLI versus SAVE-enhanced inputs on an external validation cohort

Methods	mIOU (%)	mPA (%)	Precision (%)	Recall (%)	Specificity (%)	F1-score (%)	MCC (%)
WLI	70.0 ± 1.2	81.5 ± 1.3	72.5 ± 1.3	90.3 ± 1.3	75.1 ± 1.2	80.4 ± 1.4	70.8 ± 1.4
SAVE	72.2 ± 1.1	82.3 ± 1.2	77.1 ± 1.2	88.2 ± 1.2	76.6 ± 1.2	82.3 ± 1.3	71.2 ± 1.2

This independent dataset consists of 120 images from 60 patients (16 ESCC and 44 dysplasia) at National Taiwan University Hospital (NTUH), captured using Olympus GIF-Q260 and H260 endoscopic systems.

## Discussion

**Figure 3:**
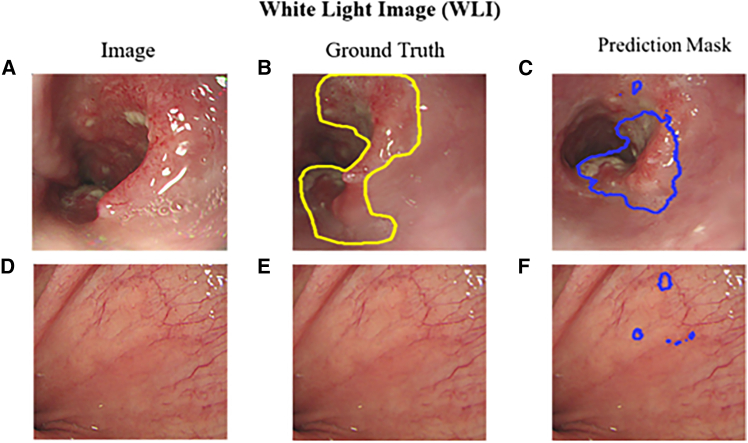
Comparison of white-light imaging (WLI) results (A) Original WLI image of a squamous cell carcinoma (SCC) case. (B) Ground truth annotation of SCC lesion in WLI image. (C) SCC lesion predicted using U-Net on WLI image. (D) Original WLI image of normal esophageal tissue. (E) Ground truth annotation for the normal case, showing no lesion. (F) Normal case predicted using U-Net, showing minimal false positives.

To compare the performance of deep learning-based lesion localization using different imaging modalities—WLI, NBI, and SAVE—we present and analyze the results illustrated in Figure 3 through 6.

### Empirical evaluation of segmentation performance in WLI and NBI modalities

**Figure 4:**
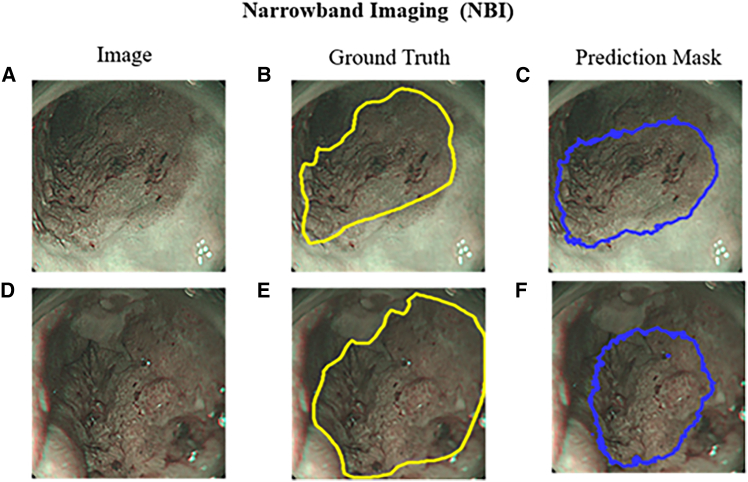
Comparison of narrow-band imaging (NBI) results (A) Original NBI image of a squamous cell carcinoma (SCC) case. (B) Ground truth annotation of SCC lesion in NBI image. (C) SCC lesion predicted using U-Net on NBI image. (D) Original NBI image of normal esophageal tissue. (E) Ground truth annotation for normal case, showing no lesion; (F) normal case predicted using U-Net, showing minimal false positives.

Figures 3 and 4 illustrate the lesion segmentation results on the traditional WLI and NBI using the U-Net deep learning model. Figure 3 presents the segmentation performance of the U-Net model on WLI. Figure 3A shows an original WLI image of an early-stage esophageal SCC case, where local erythema and tissue variation are visible but with poorly defined lesion boundaries. Figure 3B depicts the corresponding ground truth, with the lesion region manually annotated in yellow lines. In Figure 3C, the U-Net model prediction for the input image in (a) is shown in blue, indicating the segmented lesion area. Compared with (b), the model successfully captures the main lesion boundary. Figure 3D presents a WLI image of normal esophageal tissue, where the mucosal surface appears uniform with no visible abnormalities. Figure 3E illustrates the ground truth for the normal image, indicating no lesion. Figure 3F shows the model prediction for the normal image, where only minor false positives appear, demonstrating a low false positive rate and good specificity. These results indicate that despite the visual ambiguity of erythema and inflammation in WLI, the U-Net model can effectively localize lesion regions after sufficient training. The WLI model achieved an mIOU score of 69%, suggesting that even under white-light conditions, the model can provide reliable segmentation performance, particularly in SCC cases with more apparent morphological features. Figure 4 presents the results using NBI images. Figure 4A shows the original NBI image of an early-stage SCC lesion, which exhibits enhanced contrast in mucosal texture and vascular patterns. Figure 4B displays the manually annotated ground truth lesion boundary marked in yellow. Figure 4C shows the U-Net prediction in blue, which closely matches the annotated region and demonstrates excellent segmentation performance. Figure 4D is an NBI image of normal esophageal tissue, and Figure 4E shows the corresponding ground truth, indicating no lesion. Figure 4F displays the model’s prediction, which closely aligns with the ground truth and shows minimal false positives. These results highlight that the U-Net model performs better on NBI images, particularly in delineating lesion boundaries and abnormal regions. The enhanced visual contrast in NBI improves the model’s ability to distinguish inflamed or dysplastic areas. The NBI model achieved an mIOU of 71.0%, indicating superior training performance on NBI inputs compared with WLI. Overall, NBI offers clearer lesion features that can be more effectively learned and segmented by deep learning models.

### Comparative evaluation of spectral imaging approaches for early esophageal neoplasia

In evaluating the broader landscape of spectral imaging technologies for gastrointestinal neoplasia, it is evident that hardware-based spectral and hyperspectral endoscopy and computational RGB-to-spectral conversion methods each offer distinct advantages and limitations that shape their clinical applicability. Hardware hyperspectral platforms, including early polarized scanning spectroscopy systems for Barrett’s esophagus and more recent HySE-type devices, provide true pixel-wise reflectance spectra across numerous narrow wavelength bands, thereby enabling the quantitative interrogation of hemoglobin concentration, oxygenation states, and microstructural scattering properties.^38^^,^^39^^,^^40^ These systems have demonstrated the capacity to reveal dysplastic or neoplastic mucosa that remains undetectable under both high-definition WLI and NBI, and have achieved classification accuracies of approximately 80%–85% in distinguishing Barrett’s neoplasia from non-dysplastic mucosa. The ability to retrospectively reweight spectral bands to generate optimized narrow-band combinations represents an additional strength of hardware HSI, offering considerable flexibility for exploratory analyses and biomarker identification. However, clinical deployment is hindered by the need for dedicated optics and illumination modules, increased device fragility, and unavoidable trade-offs among spectral resolution, spatial resolution, and acquisition speed. The requirement for calibration and reconstruction workflows remains a substantial barrier to routine use, and motion artifacts continue to limit the feasibility of real-time hypercube acquisition during dynamic endoscopic procedures. Although conventional image-enhanced endoscopy methods such as NBI avoid these operational challenges, their reliance on only a few predefined wavelength bands cannot fully capture the spectral diversity associated with early neoplastic transformation, contributing to the persistent difficulty in detecting flat or minimally elevated lesions.

In contrast, computational RGB-to-hyperspectral and RGB-to-pseudo-NBI techniques attempt to circumvent hardware constraints by inferring virtual spectral information directly from conventional WLI data. Spectrum reconstruction algorithms, including SAVE-type methods, and deep generative models such as CycleGAN-based WLI-to-synthetic-NBI frameworks, generate pseudo-spectral images that emulate the contrast properties of hardware NBI or HSI systems.^31^^,^^41^^,^^42^ Because these approaches require no modification to clinical endoscopes and can be applied retrospectively to existing image archives, they offer a practical and scalable means of augmenting spectral content for downstream computer-aided detection. Their compatibility with deep learning models further facilitates integration into automated diagnostic pipelines. Nevertheless, reconstructed spectral signatures remain indirect estimates constrained by the limited information content of RGB signals and by the characteristics of the training data. As a result, errors in spectral recovery may be magnified when algorithms encounter data from different illumination sources, endoscope models, or patient populations. Moreover, most studies leveraging virtual spectral images or HSI-derived spectra have focused primarily on frame-level or region-level classification tasks, providing proof-of-concept improvements but falling short of delivering reliable, pixel-wise segmentation of early esophageal neoplasia under realistic clinical variability. Within this context, the SAVE-based strategy used in the present study aims to merge the workflow simplicity of conventional WLI with the enriched contrast of diagnostically relevant spectral components, thereby narrowing the gap between hardware HSI’s rich optical information and the practical requirements of routine endoscopic care. The integration of these converted hyperspectral representations with a U-Net segmentation model offers a comprehensive framework that not only enhances visual contrast but also supports precise lesion delineation, addressing a central unmet need in early esophageal cancer detection.

### Validity and uncertainty of spectral reconstruction

As mentioned in our methodology, the transformation from RGB to HSI is an underdetermined problem. Since direct validation against ground-truth spectral measurements is constrained by the proprietary nature of clinical NBI systems, the “Pseudo-HSI” generated by our model serves primarily as a feature-enhanced representation rather than a physically absolute spectral measurement. Despite this limitation, the validity of these reconstructed features is supported by their downstream utility. The significant improvement in segmentation performance (as evidenced by higher Dice and IoU scores compared to RGB baselines) indicates that the Pseudo-HSI captures diagnostically relevant structural and biochemical information that is otherwise latent in standard images. Regarding the uncertainty inherent in spectral reconstruction, we acknowledge that this process may introduce spectral noise or ambiguity. However, in our framework, this uncertainty is mitigated by the end-to-end training of the segmentation network. The deep learning model effectively learns to weigh the reconstructed spectral features, utilizing consistent patterns that correlate with lesion morphology while suppressing inconsistent reconstruction noise. Future studies incorporating phantom-based spectral measurements could further quantify this reconstruction fidelity.

### Enhancing the potential of U-Net models for esophageal lesion detection using SAVE

**Figure 5:**
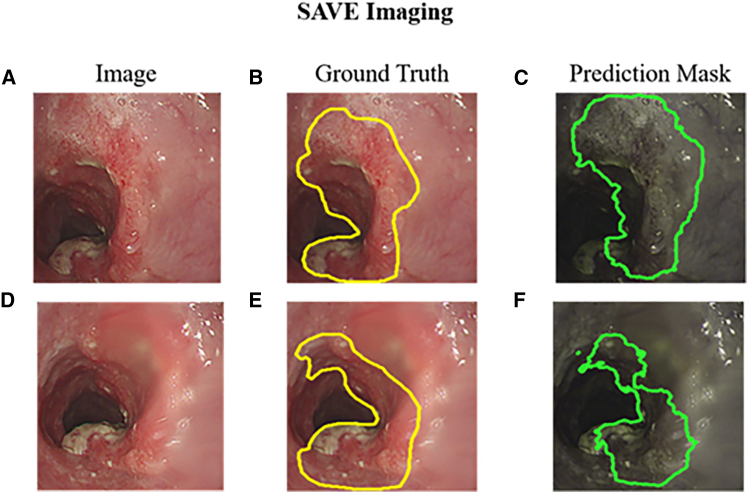
Comparison of SAVE image results (A) Original SAVE image of a squamous cell carcinoma (SCC) case, generated via hyperspectral simulation. (B) Ground truth annotation of SCC lesion in the SAVE image. (C) SCC lesion predicted using U-Net on the SAVE image. (D) Original SAVE image of normal esophageal tissue. (E) Ground truth annotation for the normal case, showing no lesion. (F) Normal case predicted using U-Net, demonstrating high specificity and minimal false positives.

**Figure 6:**
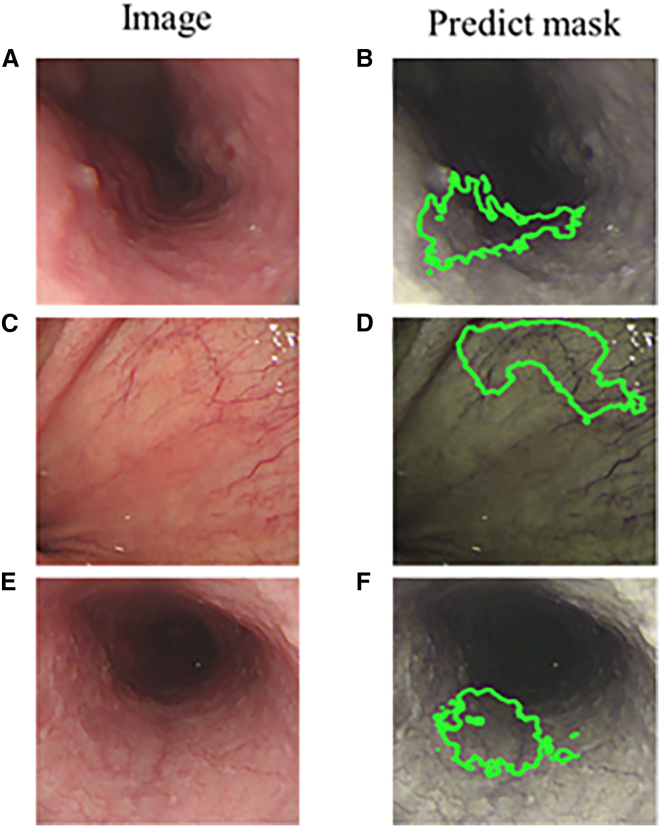
Detection results of early esophageal cancer cases (A) Original image of the first early esophageal cancer case. (B) Lesion area for the first case predicted using U-Net. (C) Original image of the second early esophageal cancer case; (D) lesion area for the second case predicted using U-Net. (E) Original image of the third early esophageal cancer case. (F) Lesion area for the third case predicted using U-Net.

SAVE was applied to convert WLI images into NBI-like simulations by using an HSI algorithm. Subsequently, a U-Net model was trained with the resulting data to further enhance lesion detection. As shown in Figure 5A, the simulated SAVE image of an SCC lesion preserves the original tissue texture of the WLI while emphasizing spectral features. Figure 5B presents the manually annotated lesion area, outlined in yellow to indicate the ground truth. Figure 5C shows the lesion region predicted by the U-Net model, marked with a green box. Compared with the ground truth, the prediction exhibits high spatial consistency and well-defined lesion boundaries. Figure 5D illustrates a normal SAVE image with clearly defined mucosal patterns and no visible abnormalities. Figure 5E is the corresponding manual annotation, confirming the absence of lesions. Figure 5F displays the model’s prediction, which aligns closely with the actual non-lesion region and demonstrates a very low false positive rate. These findings, particularly in Figures 5C and 5F, indicate that the model trained with SAVE images more accurately predicts lesion boundaries than the models trained with traditional WLI or NBI. The mIOU for SAVE-based predictions reached 74.3%, surpassing the performance of WLI and NBI models. This finding confirms the efficacy of SAVE in mimicking NBI characteristics and enhancing the deep learning model’s ability to detect early esophageal lesions. It may appear counterintuitive that the SAVE method, derived from WLI, achieved slightly higher segmentation performance than physical NBI. This can be attributed to the nature of deep learning feature extraction. While physical NBI provides superior vascular contrast, it often suffers from reduced illumination and lower signal-to-noise ratios compared to WLI. SAVE leverages the high spatial resolution, brightness, and texture richness of the original WLI while computationally enhancing the contrast of lesion areas. Essentially, SAVE provides the segmentation network with “the best of both worlds”: the structural clarity of WLI and a statistically enhanced contrast that mimics the saliency of NBI, thereby facilitating more precise boundary delineation. Figure 6 further demonstrates the performance of the U-Net model on early esophageal cancer detection by using SAVE images. Figure 6A shows an early-stage esophageal cancer image with mild erythema and subtle texture changes, making it difficult to delineate lesions by eye. Figure 6B shows the corresponding SAVE prediction, where the green contour effectively outlines the suspected lesion region. Figure 6C presents a second early-stage image with a flat appearance and no apparent abnormalities, representing a highly challenging case. However, the model successfully predicted a plausible lesion area, as shown in Figure 6D, highlighting its potential when aided by the hyperspectral data. Figure 6E shows a third early-stage esophageal image with uniform mucosa and no visible lesions. However, the model’s prediction in Figure 6F identified a potential abnormal region, indicating its sensitivity to subtle pathological features.

**Figure 7:**
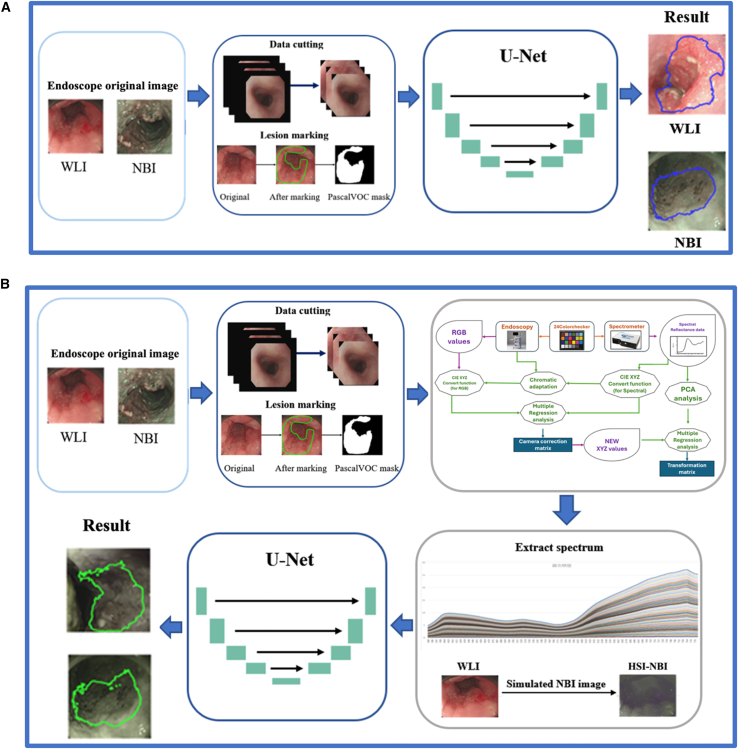
Experimental workflow for conventional and hyperspectral lesion segmentation (A) Workflow for conventional white-light imaging (WLI) and narrow-band imaging (NBI) datasets, including preprocessing, U-Net architecture training, and semantic segmentation output. (B) Workflow for the spectrum-aided vision enhancement (SAVE) framework. This process integrates spectral reconstruction from RGB values and 24-color checker calibration to generate pseudo-hyperspectral images for semantic segmentation. The dataset was split at the patient level (*n* = 99 patients for training, *n* = 25 patients for testing) to prevent data leakage.

**Figure 8:**
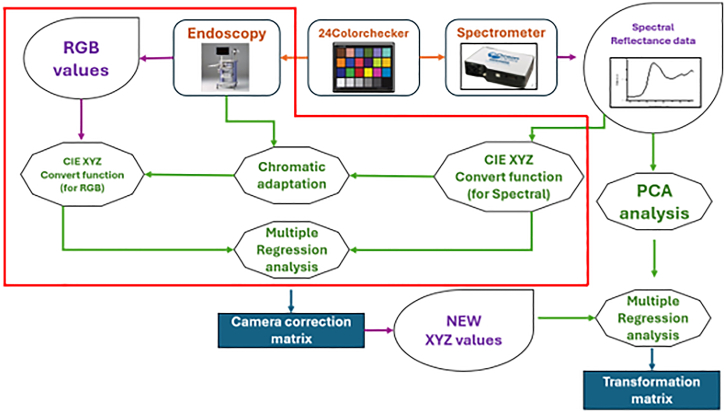
Hyperspectral conversion workflow Endoscopic images are converted into 401-band visible spectrum data by using the standard 24 Color checker (X-Rite Classic, 24 Color Checkers) as a common reference object between the endoscope and the spectrometer.

**Figure 9:**
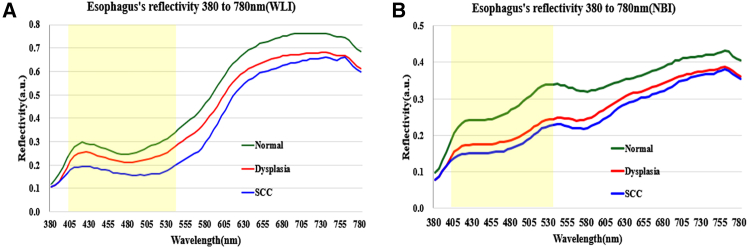
Spectral curves of normal mucosa and neoplastic lesions extracted from the computationally reconstructed pseudo-hyperspectral images Note that these spectra are derived from the SAVE reconstruction algorithm and are not direct physical measurements from a hyperspectral camera. (A) Spectral profiles of normal mucosa, dysplasia, and squamous cell carcinoma (SCC) generated from pseudo-hyperspectral reconstructions of white-light endoscopy (WLI) images. (B) Corresponding pseudo-hyperspectral profiles derived from narrow-band imaging (NBI) images. The green curve represents normal tissue, the red curve represents dysplasia, and the blue curve represents SCC. These reconstructed spectral differences informed the selection of the 415–540 nm range as the basis for generating SAVE images.

**Figure 10:**
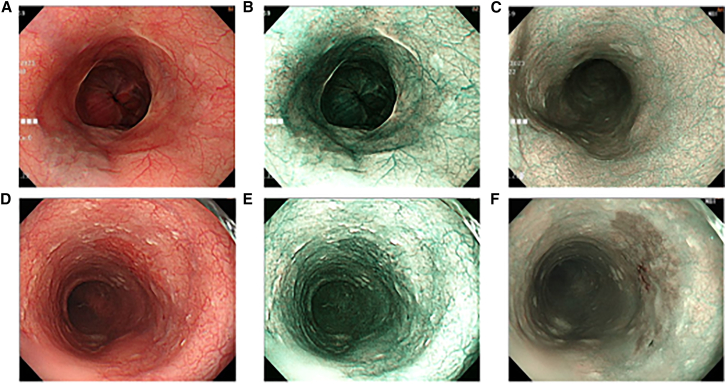
Comparison of white-light imaging, spectrum aided vision enhance, and narrow-band imaging images (A) Original white-light endoscopic image of dysplasia from patient A. (B) SAVE image corresponding to (A), generated using hyperspectral enhancement. (C) Actual Olympus narrow-band imaging (NBI) endoscopic image corresponding to (A). (D) Original white-light endoscopic image of early esophageal cancer from patient B. (E) SAVE image corresponding to (D), showing enhanced contrast and lesion boundaries. (F) Actual NBI image corresponding to (D), captured by Olympus endoscope.

In contrast to earlier SAVE studies that primarily emphasized accurate spectral reconstruction,^41^ colorimetric calibration, and variance-based band selection, the present work introduces several methodological and clinically oriented extensions (Figure 7) that substantially broaden the utility of SAVE in the computer-aided analysis of esophageal lesions. Rather than limiting band selection to global variance criteria, we reconstruct a complete 401-band hyperspectral cube (380–780 nm) for each endoscopic image (Figure 8) and perform lesion-specific spectral analysis across normal mucosa, dysplasia, and squamous cell carcinoma (Figure 9). This analysis demonstrates that the 415–540 nm range provides the strongest diagnostic contrast, enabling a pathology-guided band-selection strategy that differs fundamentally from the variance-driven approach used previously. To further align the hyperspectral reconstruction with the optical principles of clinical NBI, we extract the 415 nm and 540 nm bands, which correspond to the narrow illumination peaks of commercial NBI systems, and use them to synthesize NBI-equivalent pseudo-hyperspectral images directly from the reconstructed cube (Figure 10). These images reproduce the depth-resolved vascular contrast that underlies NBI’s diagnostic sensitivity, a capability not present in prior SAVE implementations. Finally, SAVE is integrated into a comprehensive analytical framework that couples deep-learning-based semantic segmentation with spectral-distribution modeling, thereby transforming SAVE from a reconstruction-oriented technique into a central component of a computer-aided diagnostic workflow designed to enhance the visualization and detection of early esophageal neoplasia. It is worth noting that while 577 nm represents another major hemoglobin absorption peak, it was not included in this study. This is because the 577 nm band offers similar tissue penetration depth and absorption properties to the 540 nm band (both within the Q-band region). Consequently, including 577 nm would likely introduce spectral redundancy without significantly enhancing the differential contrast between superficial and submucosal vessels, which is the primary mechanism for visualizing early esophageal lesions.

### Distinction between segmentation and clinical diagnosis

It is important to distinguish the contributions of this study from clinical diagnostic efficacy. Our results demonstrate that the SAVE framework significantly improves pixel-level segmentation performance and boundary delineation on retrospectively annotated images. While accurate segmentation is a prerequisite for computer-aided diagnosis (CADx), this study does not directly evaluate the impact of the system on clinical decision-making or real-time detection rates by endoscopists. Future prospective studies are needed to determine whether this improved segmentation capability translates into a tangible reduction in miss rates or enhanced diagnostic confidence in a clinical setting.

### Balancing recall and precision for clinical applicability

Our results demonstrate a high recall (91.2%) but a relatively lower precision (80.5%), indicating a tendency toward over-segmentation. In the context of screening for early esophageal neoplasia, high recall is clinically prioritized to minimize the risk of missing subtle, high-risk lesions (false negatives). However, excessive false positives can lead to operator fatigue and unnecessary biopsies. To address this, we propose and have preliminarily evaluated a post-processing strategy to enhance practical relevance. This includes: 1. morphological filtering: applying connected component analysis (CCA) to remove small, isolated prediction artifacts (e.g., regions <50 pixels) that are unlikely to be true lesions. 2. Confidence Thresholding: Filtering out pixels where the model’s prediction probability is below a stricter confidence threshold (e.g., 0.6 instead of 0.5). Our preliminary analysis suggests that integrating these simple post-processing steps can effectively reduce background noise and improve precision without significantly compromising the high sensitivity required for early detection. Future clinical implementations will likely incorporate these tunable parameters to allow endoscopists to adjust the sensitivity-specificity balance according to the clinical scenario.

### Model-agnostic enhancement

A critical finding of this study is that the SAVE framework functions as a model-agnostic enhancement module. Our experiments with DeepLabv3+^48^ and SegFormer^49^ confirm that the benefits of pseudo-spectral reconstruction are not limited to a specific architecture. Regardless of whether the downstream network utilizes Atrous spatial pyramid pooling (DeepLabv3+) or Self-Attention mechanisms (SegFormer), the enhanced feature space provided by SAVE consistently leads to superior segmentation accuracy compared to standard WLI. This suggests that SAVE addresses the fundamental limitation of low contrast in esophageal lesions at the data level, providing universally higher-quality input for AI analysis. Our ablation study further clarifies the mechanism of improvement. Simple contrast enhancement techniques such as CLAHE, while helpful, often amplify background noise along with the signal. In contrast, SAVE leverages the physical correlation between RGB and hyperspectral data to reconstruct specific spectral bands associated with hemoglobin absorption. This targeted enhancement allows the model to distinguish neoplastic tissue from inflamed or normal mucosa more effectively than generic image processing methods. This confirms that the performance gain is driven by the recovery of diagnostic spectral features rather than mere global contrast adjustment.

The potential for further refinement of the SAVE pipeline is highlighted by recent cross-domain advances in high-dimensional data analysis. For instance, in the field of remote sensing, Yan et al. (2026) demonstrated that a modified hierarchical vision transformer could effectively capture long-range dependencies for soil moisture retrieval. Similarly, the 401-band pseudo-HSI generated by SAVE provides a rich, high-dimensional feature space that characterizes subtle mucosal variations. While our preliminary evaluation using SegFormer showed promising gains in precision, future iterations of the SAVE framework could integrate hierarchical attention mechanisms to better model the global relationship between distant pixels and their corresponding spectral fingerprints.^50^ This synergy between physics-based spectral reconstruction and attention-based hierarchical learning represents a promising frontier for computer-aided diagnosis of early esophageal lesions.

### Cross-center validation and generalizability

The generalizability of the SAVE framework was further substantiated through cross-center validation using a completely independent dataset from National Taiwan University Hospital (NTUH). Despite the inherent variability in clinical environments and hardware configurations—specifically the use of Olympus GIF-Q260 and H260 systems—the SAVE framework maintained superior performance over standard WLI, achieving an mIoU of 72.2% compared to 70.0% for WLI.

Although a marginal performance gap exists between the external cohort and the internal test set (mIoU 72.2% vs. 74.3%), such a decrease is an expected observation in multi-center AI evaluations and remains within a clinically acceptable range for reliable lesion localization. This consistent improvement across different institutions and endoscope models suggests that the pseudo-spectral features reconstructed by SAVE address the fundamental challenge of low lesion contrast at the data level, rather than overfitting to specific center-dependent image characteristics. These findings reinforce the robustness of the SAVE framework as a scalable and model-agnostic enhancement tool for enhancing computer-aided detection in routine endoscopic practice.

### Synergistic application of deep learning and HSI in early diagnosis of esophageal cancer

The following conclusions were drawn on the basis of the findings: (1) In terms of imaging modality comparison, the traditional WLI presents certain challenges in accurately predicting lesion locations, especially in cases where inflammation and redness are not clearly visible. The U-Net model demonstrated an ability to detect lesion regions. However, its average segmentation accuracy (mIOU) was approximately 69%. By contrast, NBI provided higher visual contrast, particularly in mucosal textures and vascular distribution. The U-Net model applied to NBI images achieved better segmentation performance, with an mIOU of 71.0%. Using HSI techniques to simulate NBI-like features from WLI enhanced the spectral characteristics. The U-Net model trained on these SAVE images achieved a higher accuracy, reaching an mIOU of 74.3%. (2) In terms of improving deep learning model performance, training the U-Net model with SAVE-generated images allowed for more effective simulation of NBI spectral properties and enhanced the model’s ability to identify early lesions. With the aid of hyperspectral information, the deep learning model was able to highlight potential abnormal areas even when no obvious visual features were present, demonstrating its sensitivity to subtle pathological changes. The combination of SAVE and deep learning effectively improved the diagnostic accuracy. Future collaboration with clinicians is essential to further evaluate the model’s clinical reliability. The application of these technologies contributes significantly to early esophageal cancer detection, particularly in difficult-to-identify early-stage cases, by providing clearer lesion localization. These results indicate that integrating HSI with deep learning models can markedly enhance the detection of early esophageal cancer lesions, offering a powerful assistive tool for clinical diagnosis.

### Clinical trade-off: Sensitivity vs. precision

While our model achieves a high recall of 91.2%, the precision of 80.5% implies a certain rate of false positives. In the context of clinical screening for early esophageal cancer, we argue that high sensitivity is the paramount metric, as the cost of a missed diagnosis (false negative) significantly outweighs the inconvenience of a false alarm. We envision the SAVE framework operating as a “red flag” support tool that draws the endoscopist’s attention to subtle mucosal changes. The physician then acts as the final gatekeeper, verifying the AI-suggested regions using advanced modalities such as NBI or magnifying endoscopy before deciding on a biopsy. Consequently, the false positives generated by the model are likely to result in a brief, prolonged inspection rather than unnecessary invasive procedures. Furthermore, compared to the baseline WLI model (precision 74.9%), the SAVE framework demonstrates a substantial improvement in suppressing background noise, thereby effectively reducing operator fatigue and potential distraction in a real-world clinical workflow.

This study demonstrates that the virtual hyperspectral reconstruction of conventional endoscopic images can provide enriched spectral information that modestly improves deep-learning segmentation of esophageal lesions. By converting WLI and NBI images into 401-band pseudo-hyperspectral representations and synthesizing NBI-like images based on diagnostically relevant wavelengths, the SAVE framework enhances vascular and mucosal contrast in a manner that supports more consistent boundary identification. Models trained with SAVE-derived images achieved a 5.3% improvement in mIoU, accompanied by corresponding gains in precision and recall, indicating that the incorporation of pseudo-spectral information can benefit pixel-level lesion delineation. While exploratory in nature, these findings illustrate the potential value of integrating virtual spectral enhancement with deep-learning segmentation and highlight an emerging direction for computer-aided analysis in esophageal imaging.

### Limitations of the study

A number of limitations should be acknowledged when interpreting the findings of this study. First, a fundamental limitation is the ill-posed nature of reconstructing high-dimensional spectral data from trichromatic RGB inputs due to metamerism. The 24-patch color checker used for model construction differs from biological tissue in terms of scattering and absorption properties. Consequently, the ‘reconstructed’ spectra are mathematical approximations. Although the pseudo-hyperspectral representations provide useful spectral diversity for exploratory analysis, they remain indirect estimates derived from RGB inputs and are therefore constrained by the information content and illumination characteristics of the original images. However, our results demonstrate that these pseudo-spectral features provide sufficient contrast enhancement to significantly improve the performance of the semantic segmentation model compared to standard RGB or NBI inputs. Second, the dataset size remains modest, although this study included 60 patients from National Taiwan University Hospital (NTUH) for external validation, which initially confirmed the cross-center generalization ability, a larger-scale multicenter prospective study is still needed in the future to further confirm the real-time detection rate of the system in different clinical settings. False-positive predictions, particularly small peripheral activations, remain a notable challenge, and the lack of geometric calibration in standard endoscopy precludes retrospective measurement of lesion size or false-positive areas in absolute units. Third, the study was not designed to evaluate diagnostic outcomes such as early cancer detection or clinical decision support, and the improvements observed should be interpreted solely in terms of segmentation behavior within the available dataset. Future work will incorporate calibrated endoscopic systems or reference phantoms for spatial measurement, expand the dataset with additional institutions and imaging conditions, and integrate post-processing strategies—such as NMS, confidence mapping, and false-positive filtering—to enhance robustness and clinical applicability.

## Resource availability

### Lead contact

Further information and requests for resources and reagents should be directed to and will be fulfilled by the lead contact, Hsiang-Chen Wang (hcwang@ccu.edu.tw).

### Materials availability

This study did not generate new unique reagents.

### Data and code availability

• Data: The quantitative performance metrics and statistical data supporting the findings of this study are included within the article and its supplemental information. The raw endoscopic images are not publicly deposited due to institutional policies regarding patient privacy and Institutional Review Board (IRB) restrictions.
• Code: The reconstruction framework and core algorithms described in this study are proprietary and contain commercially sensitive technology. However, the model architecture (U-Net) and hyperparameters required to replicate the segmentation results are fully described in the STAR Methods and key resources table.
• Inquiry: Any additional inquiries regarding data access or technical implementation should be directed to the lead contact. Access to de-identified datasets may require a formal Data Use Agreement (DUA) and approval from the contributing hospitals (KMUH/NTUH).

## Acknowledgments

This research received support from the 10.13039/100020595National Science and Technology Council, Republic of China through the following grants: NSTC 109-2314-B-037-033, 110-2314-B-037- 099, 113-2221-E-037-005-MY2, and 113-2221-E-194-011-MY3. Additionally, financial support was provided by Kaohsiung Medical University Hospital research project (KMUH114-4R02), Biomedical Artificial Intelligence Academy of Kaohsiung Medical University (KMU-TC114A205-1), the 10.13039/501100012542Dalin Tzu Chi Hospital, Buddhist Tzu Chi Medical Foundation-National Chung Cheng University Joint Research Program and Kaohsiung Armed Forces General Hospital Research Program KAFGH_D_113036, Research Center on Artificial Intelligence and Sustainability, National Chung Cheng University, Taiwan under the research project grant titled “Generative Digital Twin System Design for Sustainable Smart City Development in Taiwan.”

## Author contributions

Conceptualization, Y.-K.W. and H.-C.W.; Data curation: Y.-K.W., K.-H.L., and H.-C.W.; formal analysis: Y.-K.W., C.-H.S., and H.-C.W.; funding acquisition: C.-Y.W., I.-C.W., and H.-C.W.; investigation: K.-H.L. and I.-C.W.; methodology: K.-H.L. and C.-L.C.; project administration: H.-C.W.; resources: K.-H.L., C.-H.S., and H.-C.W.; software: C.-L.C. and C.-Y.W.; supervision: I.-C.W. and H.-C.W.; writing – original draft: C.-H.S., C.-L.C., and C.-Y.W.; writing – review and editing: Y.-K.W., C.-Y.W., W.-C.C., and H.-C.W.

## Declaration of interests

Hsiang-Chen Wang from Hitspectra Intelligent Technology Co., Ltd. The authors declare that they have no conflicts of interest.

## STAR★Methods

### Key resources table

**Table:** 

REAGENT or RESOURCE	SOURCE	IDENTIFIER
**Deposited data**		

Internal Esophageal WLI/NBI Dataset (KMUH)	This study	N/A
External Esophageal WLI/NBI Dataset (NTUH)	This study	N/A

**Software and algorithms**		

PyTorch	PyTorch	https://pytorch.org/; RRID:SCR_016012
U-Net	Ronneberger et al.,^46^	RRID:SCR_017401
DeepLabv3+	Chen et al.^50^	N/A
SegFormer	Xie et al.^51^	N/A
SAVE (Spectrum-Aided Vision Enhancement)	This study	N/A

**Other**		

Olympus GIF-Q260/H260 Endoscope	Olympus	RRID:SCR_018510 (Olympus Corp.)
X-Rite Classic 24 Color Checker	X-Rite	N/A
Ocean Optics QE65000 Spectrometer	Ocean Optics	RRID:SCR_023348 (Ocean Insight)

### Experimental model and study participant details

#### Human subjects

All endoscopic images used in this study were collected from 124 patients at Kaohsiung Medical University Hospital and included 41 cases of esophageal squamous cell carcinoma (ESCC), 34 cases of high-grade or low-grade dysplasia, and 49 cases of endoscopically normal mucosa. A total of 124 patients were included, among whom 20 had early esophageal cancer, accounting for 16.1%. After exclusion of images that were unlabeled, blurry, out of focus, or obscured by mucus or inadequate insufflation, a total of 531 qualified images remained, comprising 320 WLI and 211 NBI images. For analytic purposes, these images were categorized into two groups—esophageal abnormal (dysplasia or cancer) and esophageal normal—yielding 190 abnormal and 130 normal images in the WLI set, and 81 abnormal and 130 normal images in the NBI set. To prevent data leakage and ensure unbiased evaluation, the dataset was split at the patient level. The 124 patients were randomly assigned to the training set (*n* = 99) and testing set (*n* = 25). All images derived from a single patient were contained exclusively within one set, ensuring that the model was evaluated on strictly unseen patients.

#### Ethics statement

This study adhered to the guidelines outlined in the Declaration of Helsinki and received approval from the Institutional Review Board (IRB) of Kaohsiung Medical University Chung-Ho Memorial Hospital (KMUHIRB-E(I)-20230200) and National Taiwan University Hospital (202304031DIFD). Written informed consent was waived because of the retrospective, anonymized nature of the study design.

### Method details

#### Ground truth annotation protocol

Ground-truth annotation of lesion boundaries was performed by the first author, Dr. Yao-Kuang Wang, an attending gastroenterologist with extensive expertise in the endoscopic diagnosis of early esophageal neoplasia. All images were fully de-identified in accordance with institutional review board regulations (KMUHIRB-E(I)-20230200); consequently, no patient-level clinical or pathological information was accessible at the time of annotation. The annotator was therefore aware of the overall study objective but was blinded to the definitive diagnosis for each individual case. Lesion boundaries were delineated according to established endoscopic criteria for early squamous neoplasia, including focal abnormalities in mucosal color, surface texture, vascular pattern, and the presence of a clear demarcation line under WLI and NBI. To ensure the reliability of the ground truth labels and minimize inter-observer bias, a rigorous multi-stage annotation protocol was implemented involving three senior gastroenterologists (Y.K.W., C.Y.W., and I.C.W.). Crucially, all endoscopic images included in this study were strictly correlated with their corresponding histopathological reports, with ground truth boundaries defined based on confirmed neoplastic areas from biopsy or endoscopic resection specimens. The initial delineations were performed by the first author (Y.K.W., an experienced gastroenterologist) and were subsequently reviewed independently by a senior expert (I.C.W., Chief of Gastroenterology). Any discrepancies regarding lesion boundaries were resolved through joint discussion to reach a consensus. This comprehensive workflow ensures that the segmentation masks are not only clinically consistent but also pathologically verified.

In this study, the term early esophageal squamous cell carcinoma (early ESCC) follows the American Joint Committee on Cancer (AJCC) 7th edition definition, which classifies early ESCC as disease confined to the mucosa or submucosa (T1 stage), regardless of lymph-node status. Specifically, T1a lesions are limited to the mucosa, and T1b lesions extend into but not beyond the submucosa. Because conventional gastrointestinal endoscopy systems do not encode geometric calibration or pixel-to-millimeter conversion during image acquisition, spatial measurements cannot be derived directly from WLI or NBI frames. As a result, true physical scale bars could not be generated for the images used in this study.

#### Pseudo-hyperspectral reconstruction (SAVE)

Figure 7 illustrates the experimental workflow of this study. In the process shown in Figure 7A, original endoscopic images (including WLI and NBI) first undergo data segmentation and lesion annotation to generate pixel-wise masks as ground truth labels. These labeled images are then input into the U-Net deep convolutional neural network model for semantic segmentation of lesion regions. U-Net consists of a contracting path for capturing context through 3 × 3 convolutional layers and max pooling for encoding, and an expansive path that performs upsampling and concatenation with corresponding feature maps to enable precise spatial localization and reconstruction. Its U-shaped architecture gives the model its name “U-Net.” The fundamental design of U-Net is derived from the traditional autoencoder structure, composed of an encoder and a decoder, allowing the model to compress and reconstruct data while preserving semantic consistency. In this study, the U-Net model was implemented using the Pascal VOC format and the PyTorch framework. After annotation, all images were resized to 224 × 224 and compressed through four pooling layers to extract hierarchical lesion features, resulting in feature maps of sizes 112 × 112, 56 × 56, 28 × 28, and 14 × 14. The extracted feature maps were then progressively upsampled via transposed convolution to reconstruct the output image at 224 × 224 resolution. This architecture effectively captures global semantics and local details, improving lesion localization accuracy and yielding clear segmentation contours for WLI and NBI images. Figure 7B continues with the same preprocessing and lesion annotation steps but replaces the U-Net segmentation with a spectral information modeling process. In this approach, endoscopic images are transformed into multiple color spaces (e.g., RGB, HSV, and YCrCb), and features are extracted from individual channels. The extracted color-space features are then combined with simulated spectral data for classification and analysis, constructing a spectral distribution model corresponding to different lesion types. This model integrates conventional color features with simulated wavelength responses, enhancing the sensitivity and specificity of lesion identification, and provides an alternative, non-deep-learning-based method for assisted image analysis.

First, a data-driven hyperspectral reconstruction method was used to convert endoscopic images obtained under WLI and NBI into spectral data comprising 401 bands from 380 to 780 nm. The overall image transformation workflow is illustrated in Figure 8. A standard 24-color checker (X-Rite Classic, 24 Color Checkers) was used as a common reference target between the endoscopic camera and the spectrometer to construct the conversion model. In this process, the sRGB values of the endoscopic images were first normalized and linearized by inverting the standard sRGB gamma function, and then converted to CIE 1931 XYZ coordinates. To ensure that the XYZ values accurately reflected the illumination and spectral sensitivity of the specific endoscopic camera, a camera correction matrix was estimated by least-squares regression using paired measurements from the 24-patch Macbeth Color Checker. This calibration also incorporated third-order polynomial terms to compensate for sensor nonlinearity. The resulting matrix enabled the corrected XYZ values of the endoscopic images to match the XYZ values derived from spectrometer-measured reflectance spectra of the same patches. Simultaneously, the reflectance spectra of all 24 color patches were measured with a spectrometer at 1-nm intervals from 380 to 780 nm and assembled into a 401 × 24 reflectance matrix. Principal component analysis (PCA) was then applied to derive a low-dimensional spectral basis that captured more than 99% of the total variance. Each reference spectrum was represented within this basis by a PCA score vector, enabling a compact yet faithful reconstruction of the underlying spectral signatures. A linear regression model was subsequently constructed to link the corrected XYZ values of each color patch to its corresponding PCA score vector. This regression matrix enabled the projection of any new endoscopic pixel from XYZ space into the PCA coefficient space. For each pixel in a white-light endoscopic image, the normalized and linearized sRGB values were transformed into corrected XYZ coordinates, mapped to PCA scores via the regression model, and finally reconstructed into a full 401-band reflectance spectrum using the PCA loading matrix. Applying this procedure to every pixel yielded a pseudo-hyperspectral cube in which each pixel was associated with an estimated reflectance curve spanning the visible spectrum. The information from both sources was ultimately merged to construct a transformation matrix capable of converting the endoscopic images into hyperspectral data with 401 spectral bands. This hyperspectral data provides pixel-wise spectral characteristics across the visible range (380–780 nm). The spectral reflectance of annotated lesion areas and normal tissue was further analyzed and compared, as shown in Figure 9. Figure 9A displays the reflectance distribution across 380–780 nm for three tissue types—dysplasia, SCC, and normal tissue—under WLI. Figure 9B shows the corresponding spectral distribution under NBI. The reflectance differences among tissue types are most pronounced in the spectral range between 415 and 540 nm. Therefore, this range was selected as the key feature band for subsequent analysis. The selected spectral band was compressed into three channels by using PCA to further reduce data dimensionality while retaining the main variance features, allowing it to be used in subsequent image analysis and model training.

After the hyperspectral image conversion modeling was completed, the original endoscopic sRGB images were successfully transformed into hyperspectral images covering the spectral range of 380–780 nm with 401 channels. These hyperspectral data allow each pixel to be represented by a reconstructed reflectance curve derived from a calibrated low-dimensional spectral subspace, providing spectrally meaningful pixel-wise information across the 380–780 nm range. In this study, each pseudo-hyperspectral image was reconstructed as a 401-channel spectral cube spanning the visible range, and all subsequent analyses and band selections were performed within this full 401-band dataset. Among these 401 spectral bands, the 415 nm and 540 nm wavelengths were specifically selected for reconstructing images that simulate the optical characteristics of clinical narrow-band imaging (NBI). These two wavelengths correspond directly to the narrow-band illumination peaks used in commercially available NBI systems and were therefore essential for generating pseudo-NBI images that preserve the diagnostic contrast mechanisms of true NBI. The 415 nm band lies in the short-wavelength blue region, where hemoglobin absorption is maximal and tissue penetration is minimal, enabling enhanced visualization of superficial mucosal microvasculature and early neoplastic changes. The 540 nm green band penetrates slightly deeper and accentuates subepithelial vessels while maintaining strong hemoglobin contrast. Together, these two wavelength channels leverage the complementary depth-resolved vascular information inherent to NBI, allowing the reconstructed pseudo-NBI images to enhance mucosal and microvascular detail beyond what is achievable with broadband white-light illumination. The selected bands were converted into the XYZ color space by using Equations 1, 2, and 3 and then transformed back into the RGB space via Equation 4, allowing the original white-light images to be reconstructed into simulated NBI-like images (referred to as HSI-NBI). Subsequently, chroma and luminance adjustments were applied to further enhance the simulated image quality to closely resemble actual NBI images. Figure 10 presents a comparative visualization of early esophageal dysplasia images under WLI, SAVE, and NBI modalities. Figure 10A shows a WLI image from patient A with dysplasia, where the mucosal color is relatively uniform and the contrast between vessels and lesions is unclear. Figure 10B displays the corresponding SAVE image generated using the proposed technique, which enhances hemoglobin absorption-related wavelengths (415 and 540 nm) and applies color correction to make vascular structures and mucosal boundaries more distinct. Abnormal regions are clearly highlighted, improving visual recognition. Figure 10C shows the actual NBI image of the same region, offering high-contrast vascular textures used as a reference for SAVE validation. Similarly, Figure 10D shows an early-stage esophageal cancer WLI image from patient B, where the lesion is difficult to distinguish from the surrounding tissue. Figure 10E, the corresponding SAVE image, displays enhanced lesion boundaries and improved color contrast, facilitating early lesion identification. Figure 10F shows the actual NBI image, demonstrating comparable contrast enhancement to the SAVE image. The SAVE technique proposed in this study, based on an HSI conversion algorithm, enables conventional white-light endoscopic images to be transformed into high-contrast images with NBI-like visual properties. HSI spans continuous spectral bands from 400 nm to 780 nm, markedly surpassing traditional RGB imaging in spectral resolution. By simulating hemoglobin absorption characteristics, SAVE remarkably enhances the contrast of vascular and mucosal structures, allowing early-stage lesions—such as mucosal erosion, ulceration, or neoplasia—to be clearly visualized. Experimental validation shows that the technique improves the CIE DE2000 color difference values and structural similarity index measure while reducing image entropy, effectively distinguishing between normal and abnormal tissues.

(Equation 1) $$X=k\int_{380nm}^{780nm}S\left(\lambda \right)R\left(\lambda \right)\bar{x}\left(\lambda \right)d\lambda$$

(Equation 2) $$Y=k\int_{380nm}^{780nm}S\left(\lambda \right)R\left(\lambda \right)\bar{y}\left(\lambda \right)d\lambda$$

(Equation 3) $$Z=k\int_{380nm}^{780nm}S\left(\lambda \right)R\left(\lambda \right)\bar{z}\left(\lambda \right)d\lambda$$

(Equation 4) $$\left[\begin{array}{c} R\\ G\\ B \end{array}\right]=\left[\begin{array}{ccc} 3.240479 & -1.53715 & -0.498535\\ -0.969256 & 1.875991 & 0.041556\\ 0.055648 & -0.204043 & 1.057311 \end{array}\right]\left[\begin{array}{c} X\\ Y\\ Z \end{array}\right]$$

#### External validation cohort

To evaluate the generalization ability of the SAVE framework, this study additionally included an independent external validation set from National Taiwan University Hospital (NTUH). This study was approved by the Institutional Review Board of NTUH (IRB No. 202304031DIFD), and the requirement for informed consent was waived due to the retrospective nature of the study. This dataset contained 120 independent images from 60 patients (16 with ESCC and 44 with dysplasia). Image acquisition devices included Olympus GIF-Q260 and H260 systems. All external images followed the same annotation and preprocessing workflow as the original dataset.

### Quantification and statistical analysis

In this experiment, cross entropy loss was adopted as the loss function, and the model was trained over 50 epochs. As training progressed, the loss value gradually decreased with each epoch, indicating the model’s continuous learning and optimization. Given the complexity of the training data, the initial loss was approximately 0.145, which eventually converged to 0.0185 after multiple rounds of weight updates and backpropagation. This finding demonstrates the model’s ability to effectively fit the data and accurately predict classification outcomes. On the basis of the observed training behavior and reference from previous literature, the U-Net model in this study was trained for 50 epochs.^51^ This setting sufficiently covers the convergence stage observed around epoch 40 and provides a slight redundancy buffer to ensure stable performance. Additionally, corresponding to clinical esophageal cancer clinical target volume segmentation tasks, prior studies have shown that training and validation losses tend to stabilize after epoch 40, thereby validating the robustness and appropriateness of the 50-epoch setting. For the loss calculation in the U-Net model, the cross-entropy loss function was employed to measure the difference between predicted class probabilities and actual labels. Each predicted class probability was compared against the ground truth (labeled as 0 or 1), and the discrepancy was penalized accordingly, serving as the basis for model learning. The formula consists of two main components: the positive term, which contributes when the ground truth is 1, and the negative term, which takes effect when the ground truth is 0. These two components do not act simultaneously. The cross-entropy loss is defined as follows:

(Equation 5) $$Cross\;Entropy\;Loss=\frac{-1}{N}\times \sum_{1}^{N}y_{i}*\log\left({\hat{y}}_{i}\right)+\left(1-y_{i}\right)\times \log\left(1-{\hat{y}}_{i}\right)$$
